# Peripheral Neuromuscular Fatigue Responses of the Knee Extensors to Distinct Concurrent Training Protocols: A Preliminary Study

**DOI:** 10.3390/jfmk11020181

**Published:** 2026-04-29

**Authors:** Tomás T. Freitas, Elena Marín-Cascales, Cristian Marín-Pagán, Linda H. Chung, Antonio Martínez-Serrano, Nicola A. Maffiuletti, Anthony J. Blazevich, Pedro E. Alcaraz

**Affiliations:** 1 UCAM Research Center for High Performance Sport, UCAM Universidad Católica de Murcia, 30107 Murcia, Spain; tfreitas@ucam.edu (T.T.F.); emarin@ucam.edu (E.M.-C.); cmarin@ucam.edu (C.M.-P.); lhcung@ucam.edu (L.H.C.); martinez.serrano97@gmail.com (A.M.-S.); 2 Facultad de Deporte, UCAM Universidad Católica de Murcia, 30107 Murcia, Spain; 3 NAR—Nucleus of High Performance in Sport, São Paulo 04753060, Brazil; 4 International Strength & Conditioning Society, 30008 Murcia, Spain; 5 Human Performance Lab, Schulthess Clinic, 8008 Zurich, Switzerland; nicola.maffiuletti@kws.ch; 6 Centre for Exercise and Sports Science Research, School of Medical and Health Sciences, Edith Cowan University, Joondalup 6027, Australia; a.blazevich@ecu.edu.au

**Keywords:** aerobic training, strength training, HIIT, neuromuscular function, performance impairment

## Abstract

**Background:** This study aimed to investigate the extent and time course of peripheral neuromuscular fatigue of the knee extensors following different concurrent training protocols in recreationally active men. **Methods:** In a randomized, counterbalanced, crossover design, ten participants completed one exercise session of three concurrent exercise protocols in consecutive weeks and in similar resting conditions: traditional concurrent training (TCT), sprint interval training (SIT), and high-intensity resistance circuit training (HRC). Maximal voluntary isometric contraction (MVIC) and electrically evoked tetanic force of the knee extensors were assessed before, immediately after, and at 24 and 48 h following each exercise session. Linear mixed models were used to examine the differences among exercise modalities and time points. **Results:** No significant changes were found in MVIC force following HRC and TCT at any time point (*p* > 0.05), while significant declines were observed post-exercise (*p* = 0.015), 24 h (*p* = 0.001) and at 48 h (*p* = 0.003) after SIT. Moreover, MVIC force was significantly lower for SIT than HRC at 48 h (*p* = 0.001). Tetanic force significantly declined in SIT from pre-exercise to post-exercise (*p* = 0.034), with significant differences when compared to HRC (*p* = 0.003) and TCT (*p* = 0.003). HRC and TCT induced no knee extensor fatigue, contrary to a single session of SIT. **Conclusions:** Peripheral fatigue seemed to prevail following SIT in comparison with HRC and TCT, as seen by the decreased tetanic force in the former only. From an applied perspective, practitioners should carefully plan training activities to be performed the days following a SIT session, as force-generating capacity may be impaired for up to 48 h.

## 1. Introduction

Sports performance is often determined by multiple physical qualities that must be developed concurrently [1,2]. Traditionally, the term “concurrent training” has been used to describe exercise regimens that combine resistance and endurance training within periodized strength and conditioning programs [3,4]. The main rationale for using this method is that on the one hand, resistance training promotes neuromuscular (i.e., strength- and power-related) and structural (i.e., hypertrophy) adaptations [5,6], whilst on the other hand, endurance training improves cardiac and vascular health, mitochondrial function [7], and body composition [8]. Not unexpectedly, extensive research has demonstrated that concurrent training may be beneficial to counteract disorders such as sarcopenia [9], type 2 diabetes, and obesity [10], and also improve performance in athletic populations (i.e., team-sport athletes) [11,12] and recreationally active individuals [13,14].

Despite its effectiveness, some major downsides of traditional concurrent training (TCT) have been highlighted. First, the typical duration of both single TCT sessions and training interventions is relatively long [4,14], which may negatively affect exercise adherence, including in high-performance sports contexts in which athletes have limited time to devote to strength and conditioning practices. Second, TCT interventions may result in the so-called interference effect [3,4,7,14], which can lead to compromised strength gains compared to resistance training programs performed in isolation due to both training-related [3,7] and non-training-related factors [4]. As such, investigating the effectiveness of time-efficient training methods focused on generating broad cardiovascular, metabolic, and neuromuscular adaptations whilst reducing fat mass and increasing muscle mass is of great interest.

In this respect, sprint interval training (SIT), characterized by efforts performed at intensities equal to or greater than maximal oxygen uptake (VO_2max_), including “all out” efforts [15], has been proposed as a suitable method to promote these adaptations. For instance, low-volume cycling-based SIT protocols have been found to induce similar increases in skeletal muscle oxidative capacity, metabolic adaptations during exercise, and performance improvements to traditional high-volume endurance training [16,17], supporting the notion that this type of stimulus seems to be both effective and time-efficient in healthy recreationally active individuals. A previous investigation reported that an SIT protocol performed during the preseason period in team-sport athletes resulted in positive neuromuscular adaptations (i.e., increases in maximal voluntary strength and neural drive and decreases in antagonist co-activation) [18]. Therefore, given its potential to improve important neuromuscular variables and to promote both resistance [18] and endurance training-related adaptations [15,16,17], SIT might be considered a valid concurrent training approach.

Another time-efficient method that can promote concomitant cardiovascular and neuromuscular adaptations is circuit-based resistance training [19,20]. A meta-analysis [19] reported that this training modality may be effective for improving maximal voluntary strength and maximal oxygen uptake in healthy individuals. In particular, high-intensity resistance circuit (HRC) protocols performed with loads corresponding to six repetitions maximum (RM) have been found to result in strength increases comparable to traditional heavy-load resistance training protocols in recreationally active individuals [20,21,22].

Despite the growing interest in these alternative forms of concurrent training, further research on the acute effects of TCT, SIT, and HRC protocols on neuromuscular function, including fatigue, is needed. Reductions in knee extensor maximal voluntary isometric contraction (MVIC) force and increases in rating of self-reported muscle fatigue have been reported 24 h following a TCT session [22], but the immediate effects of this exercise modality are not known. Concerning HRC, knee extensor MVIC force and resting twitch force declined to a similar extent immediately after completion of a protocol in recreationally active participants, suggesting the accumulation of peripheral fatigue [23]. However, neuromuscular changes 24 h or 48 h (i.e., residual fatigue) following exercise were not assessed, so the time course of fatigue (and recovery) after a bout of HRC remains unknown. With regard to SIT, a compelling body of evidence has reported neuromuscular function impairments following repeated-sprint protocols that were mainly due to peripheral fatigue [24,25,26]. However, the extent and time course of neuromuscular fatigue have never been compared between TCT, SIT and HRC protocols.

Based on all of the above, the main aim of the present study was to provide new information regarding the extent and time course of peripheral neuromuscular fatigue of the knee extensors following TCT, HRC, and SIT protocols in young recreationally active adults. Identifying the prevalence of knee extensor peripheral fatigue following these specific and highly used exercise modalities is of substantial interest to both practitioners and researchers, especially from a recovery perspective. Moreover, understanding the associated time course of fatigue recovery may prove valuable to exercise professionals, as it may help determine the most appropriate training program according to individual needs and contextual factors (e.g., positive or negative interference effects between training loads, sessions, or moment of the season in team-sports). It was hypothesized that SIT and TCT, but not HRC, would impair neuromuscular function, with the first exercise protocol resulting in greater peripheral fatigue.

## 2. Materials and Methods

### 2.1. Study Design

A randomized, counterbalanced, crossover study design was used to investigate the peripheral neuromuscular fatigue of the knee extensor muscles following a single bout of TCT, HRC, and SIT exercise. The main neuromuscular variables included MVIC force as a marker of maximal strength and electrically evoked tetanic force as a marker of peripheral muscle function. All outcomes were assessed before and immediately after each exercise modality, as well as 24 and 48 h after exercise. The experimental procedures were conducted over a 4-week period, with participants reporting to the laboratory once in week 1 and three times in each of the following weeks. In week 1, participants completed a familiarization session for the MVIC assessment and electrical stimulation, during which the stimulus intensity (i.e., set to a level that produced 40–50% of the individual MVIC force) was determined. Additionally, in this session the participants’ 6-RM load was determined for all the exercises in the TCT and HRC protocols and a progressive treadmill test was completed to determine each individual’s second ventilatory threshold (VT2) via the ventilatory equivalent method described by Wasserman et al. [27]. In weeks 2, 3, and 4, participants completed the TCT, HRC, or SIT protocol in the first weekly visit in a randomized order (same day of the week and same time of the day). Then, they reported to the lab at the 24 and 48 h time points (weekly visits 2 and 3, respectively) to complete the corresponding testing protocol. Participants were instructed to refrain from strenuous exercise for at least 48 h prior to the first visit. They were also asked to maintain their usual diet and to avoid alcoholic beverages on the day preceding each exercise session.

### 2.2. Participants

Ten healthy male participants (age: 24 ± 5 years; height: 175.9 ± 4.9 cm; weight: 76 ± 11 kg) that met the following inclusion criteria volunteered to participate in the study: (1) experience with resistance training, having performed resistance exercise on average 2–3 times per week most weeks in the 4 years prior to the study; (2) experience with running (endurance) training, having completed an average of 2–3 weekly sessions in the 2 years prior to the study; and (3) no musculoskeletal disorders or recent injuries. Participants were informed of the investigation procedures and signed an informed consent. The study was conducted according to the Helsinki Declaration was approved by the University’s Institutional Science Ethics Committee (CE031704).

### 2.3. Procedures

#### 2.3.1. Assessment of MVIC and Tetanic Force

Participants sat upright in a chair with the hips and the right knee flexed at 90° and the ankle strapped directly to a load cell sampling at 1500 Hz (Model SML500, Interface Inc., Scottsdale, AZ, USA) that was fixed to a custom-made apparatus. Participants first performed 3 MVIC trials of the knee extensors, each lasting ~5 s, separated by 2 min of rest. They were instructed to contract “as hard as possible” using a progressive torque buildup. To ensure effort maximality, participants were verbally encouraged and at least 2 MVICs had to be within 10% of one another. The highest MVIC force was retained.

Approximately 3 s after each MVIC trial, electrically evoked 100 Hz trains were applied to assess tetanic force. Two bipolar 10 × 15 cm stimulating electrodes were positioned proximally and distally over the quadriceps muscle belly and secured with a Velcro strap. Signal 6.0 software (Cambridge Electronic Design Ltd, Cambridge, UK) was used to control the stimulating characteristics: pulse duration of 2000 µs, frequency of 100 Hz, train duration of 500 ms (50 pulses), voltage of 400 V. Stimulus intensity was set to a level that produced 40–50% of the individual MVIC force based on the outcomes obtained in the familiarization session (used to minimize possible learning effects across the different testing sessions). To achieve this pre-established value, several trains were applied at rest while progressively increasing current intensity (5 mA steps every 2–5 s; intensity range: 20–70 mA) until the intended force level was attained. The electrical stimulator (Digitimer DS7A, Digitimer Ltd., Welwyn Garden City, UK) and the load cell receiver were synchronized using a CED Micro3 1401 data acquisition unit (Cambridge Electronic Design Ltd., Cambridge, UK). Noraxon MR 3.6.20 software (Noraxon, Scottsdale, AZ, USA) was used to record force. The MVIC-stimulation sequence was repeated 3 times, with the highest tetanic force being kept for analysis.

#### 2.3.2. Determination of the 6-RM Loads

The 6-RM load for all exercises was determined during the familiarization week. An initial resistance was selected based on each participant’s self-perceived ability to perform 6 repetitions. After two warmup sets at approximately 60% and 85% of the aforementioned load, respectively, participants executed their 6-RM attempt. Following this set, if 6 ± 1 repetitions were completed, the load was adjusted by approximately 2%, and if participants were able to complete 6 ± 2 repetitions, it was accommodated by 5% [28]. Absolute failure or technical breakdown were used as the criterion for determining the 6-RM load.

#### 2.3.3. Determination of the Second Ventilatory Threshold

An incremental test was conducted on a treadmill (Technogym Run Excite Med., Cesena, Italy) until exhaustion. Participants started walking at 4.5 km·h^−1^ for 2 min as a warmup. A stepwise maximal test was then initiated, with 1 min intervals and speed increases of 1 km·h^−1^ at each step. Respiratory gas exchange was measured using a breath-by-breath metabolic cart (Metalyzer, Cortex, Leipzig, Germany). The VT2 was established using the ventilatory equivalents method described by Wasserman [27], and was used to control training intensity based on the heart rate at which this threshold occurred.

#### 2.3.4. Exercise Protocols

**Figure 1:**
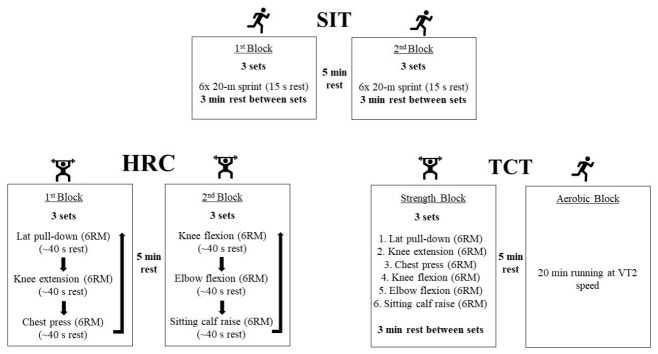
Schematic representation of the SIT, HRC, and TCT protocols. RM: repetitions maximum; VT: ventilatory threshold.

*SIT protocol.* The SIT session consisted in two blocks separated by 5 min of rest (Figure 1). Participants completed 3 sets of six 20 m all-out running sprints in each block, with 15 s of passive or slow-walking recovery between each sprint. A passive rest period of 3 min was allowed between sets. The two blocks were identical with regard to the number of sets, the repetitions performed, and the duration of the rest periods. The sprints were completed on an indoor court with constant temperature (~21 °C), and loud verbal encouragement was given throughout the exercise session. The total SIT session time was ~32 min, and the training density was 1:3 (~5 s effort; 15 s recovery).

*HRC protocol.* The HRC protocol consisted of six exercises divided in two short circuits (i.e., blocks) of three exercises, as proposed by Alcaraz et al. [29]. The first circuit included the lat pull-down, knee extension, and chest press exercises, and the second circuit included the knee flexion, elbow flexion (biceps curl), and seated calf raise exercises. Participants completed 3 sets of each circuit with the previously determined 6-RM load for every exercise. The concentric phase was performed at maximum velocity, whereas the eccentric phase was performed in a controlled manner, lasting approximately 3 s. The rest period between exercises was 40–45 s, which allowed a local muscle recovery of ~3 min (i.e., time separating a set of a given exercise and the next set of the same exercise). The second block started 5 min after the end of block 1 (Figure 1). The HRC session lasted ~45 min and the training density was 1:3 (15 s effort; 45 s recovery).

*TCT protocol.* The TCT protocol consisted of two blocks: strength and aerobic training exercise sessions (Figure 1). The strength block included the same 6 exercises performed in the HRC and consisted of 3 sets per exercise with the 6-RM loads. The TCT protocol was not completed in a circuit, as 3 min of passive rest was granted between sets. Briefly, after the completion of the 3 sets of the first exercise, participants completed the subsequent 3 sets of the following exercise. The concentric–eccentric ratio was the same as in HRC (maximum velocity: 3), and the rest period between strength and aerobic blocks was 5 min. The aerobic block comprised 20 min of treadmill running at the speed at which the second ventilatory threshold was reached, with a 1% grade. The total duration of the TCT was ~110 min.

### 2.4. Statistical Analysis

Descriptive data are presented as means ± SD. All statistical analyses were performed with the JAMOVI 2.3.21 statistical package. Linear mixed models were used to examine the effects of the different exercise modalities (TCT, HRC, and SIT) at different time points (pre-exercise, post-exercise, 24 h and 48 h). Model residuals were visually inspected to examine deviations from homoscedasticity (i.e., residuals vs. fitted values) or normality (i.e., Q–Q plots). All assumptions were met. When statistically significant effects were found, post hoc comparisons were performed using Tukey’s test. The statistical level of significance was set to *p* < 0.05. Absolute reliability was established using the coefficient of variation (CV), and relative reliability was determined with a two-way mixed-effect, absolute agreement, single-measure intraclass correlation coefficient (ICC) calculated from the pre-exercise values obtained during the first exercise week (i.e., week 2). Partial eta-squared (ηp^2^) and modified Cohen’s effect sizes (ES) and their 95% confidence limits were calculated for the linear models and pairwise comparisons, respectively, to determine the magnitude of the differences. Threshold values of ≥0.01 (small), ≥0.06 (medium), and ≥0.14 (large) were used for ηp^2^, and ≥0.2 (small), ≥0.6 (moderate), ≥1.2 (large), and ≥2.0 (very large) for modified Cohen’s ES [30].

## 3. Results

Table 1 presents the descriptive data for the different variables at each time point following the HRC, SIT, and TCT protocols.

**Figure 2:**
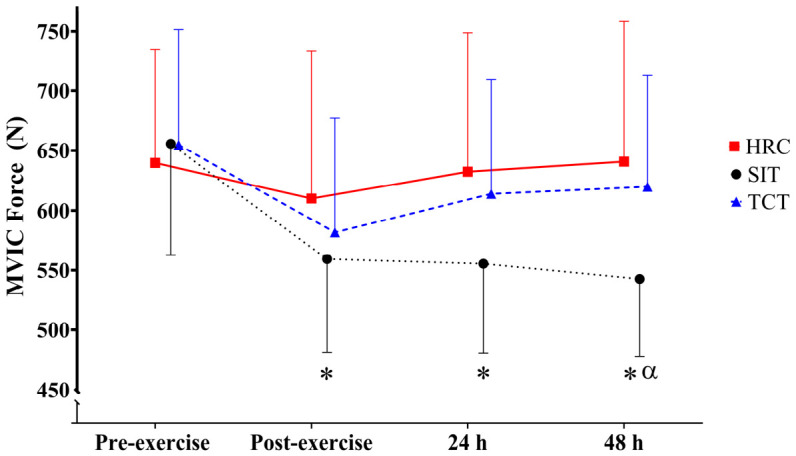
MVIC force at each time point following SIT, HRC, and TCT protocols. * Significant differences with respect to pre-exercise. α: Significant difference between SIT and HRC.

Considering MVIC force (ICC = 0.956; CV = 3.61%), significant medium group (*p* = 0.002; ηp^2^ = 0.12 [0.02; 0.24]) and time (*p* < 0.001; ηp^2^ = 0.22 [0.07; 0.34]) effects and large group*time (*p* = 0.032; ηp^2^ = 0.14 [0; 0.22]) interactions were found. HRC displayed significantly higher MVIC force values than SIT (*p* = 0.002; ES = 0.51 [0.05; 0.97]), representing a small difference irrespective of time point. No other differences were found between groups. Pre-exercise values were significantly greater than post-exercise (*p* < 0.001; ES = 0.67 [0.14; 1.20]), 24 h (*p* = 0.004; ES = 0.48 [0.06; 1.01]) and 48 h (*p* = 0.002; ES = 0.48 [0.05; 1.00]) when analyzing all groups together, with differences of small-to-moderate magnitude. Regarding group*time interaction, large significant declines in MVIC force from pre- to post-exercise (*p* = 0.015; ES = −1.08 [−2.01; −0.14]), 24 h (*p* = 0.001; ES = −1.14 [−2.08; −0.19]) and 48 h (*p* = 0.003; ES = −1.35 [−2.32; −0.38]) were observed only in SIT (Figure 2). Lastly, significant moderate differences at 48 h were observed when comparing HRC to SIT (*p* = 0.001; ES = 0.99 [0.06; 1.92]) (Figure 2). There were no other significant differences identified.

**Figure 3:**
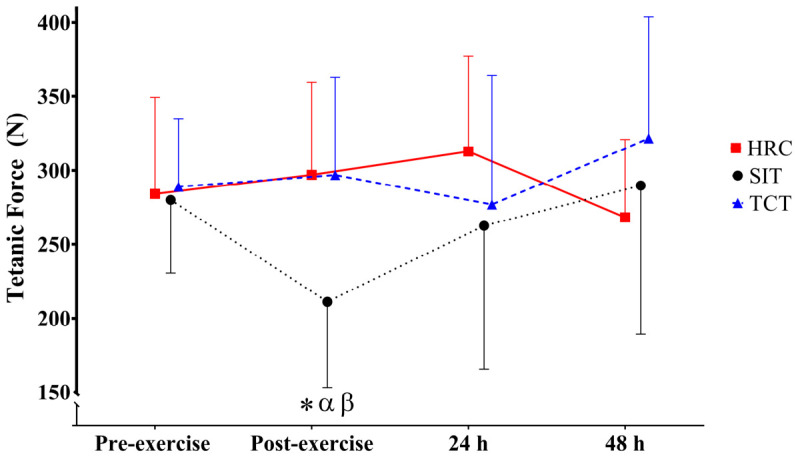
Tetanic force at each time point following SIT, HRC, and TCT protocols. * Significant differences with respect to pre-exercise. α: Significant difference between SIT and HRC. β: Significant difference between SIT and TCT.

Concerning tetanic force (ICC = 0.871; CV = 5.94%), medium group (*p* = 0.004; ES = ηp^2^ = 0.11 [0.01; 0.23]) effects and large group*time (*p* < 0.001; ηp^2^ = 0.24 [0.06; 0.34]) interactions were observed. However, no time effect was noticed (*p* = 0.129; ηp^2^ = 0.06 [0.00; 0.15]). Overall, the values in SIT were significantly lower than HRC (*p* = 0.023; ES = −0.41 [−0.87; −0.05]) and TCT (*p* < 0.005; ES = −0.46 [−0.92; −0.01]), with small ES observed. When comparing pre- to post-exercise, significant large declines in tetanic force were found in SIT (*p* = 0.034; ES = −1.23 [−2.18; −0.27]), but not in HRC (*p* = 1.000; ES = 0.15 [−0.72; 1.03]) or TCT (*p* = 1.000; ES = 0.13 [−0.74; 1.01]) (Figure 3). No differences were found at 24 h or 48 h following any of the exercise protocols. Finally, as shown in Figure 3, SIT resulted in significantly lower tetanic force than HRC and TCT post-exercise (*p* = 0.003; ES = −1.37 [−2.35; −0.40]; *p* = 0.003; ES = −1.33 [−2.29; −0.36], respectively), with differences of large magnitude identified.

A sensitivity analysis was conducted using G*Power (version 3.1) to estimate the minimum detectable ES for the present study. Given its design, in which all participants completed all experimental conditions, the analysis was conducted using a repeated-measure ANOVA (within-subject factors). The following parameters were specified: one group, three measurements (corresponding to each experimental condition), a total sample size of 10 participants, an alpha level of 0.05, and a power of 0.8. The correlation among repeated measures was set to 0.50, and the nonsphericity correction was assumed to be 1. Under these assumptions, the analysis indicated a minimum detectable ES of Cohen’s f = 0.43 (corresponding to a Cohen’s d of ~0.86). Considering that the observed ηp^2^ values for the group*time interaction were 0.14 for MVIC and 0.22 for tetanic force (corresponding to Cohen’s f values of approximately 0.40 and 0.53, respectively), the achieved statistical power was estimated to be approximately 0.72 for the former variable and 0.93 for the latter. This indicates that while the study had limited sensitivity to detect small interaction effects (e.g., for MVIC), it was adequately powered to detect moderate-to-large effects, such as those observed for tetanic force.

## 4. Discussion

The present study aimed to investigate the extent and time course of peripheral neuromuscular fatigue following three different concurrent training exercise modalities (i.e., SIT, HRC, and TCT) in recreationally active males. The main findings indicated that: (1) MVIC force was not affected by HRC or TCT, contrary to the SIT condition, after which a significant decline was observed; and (2) SIT resulted in significantly greater peripheral fatigue, as shown by the decreased tetanic force post-exercise and the significant differences with respect to the other protocols. Despite the fact that comparing protocols with such dissimilar characteristics (e.g., total duration and mechanical work, muscle involvement, metabolic stress, or time under load) can be challenging and definitely warrants caution when interpreting the results, particularly as the observed differences could reflect differences in the aforementioned factors rather than protocol structure per se, the findings herein provide practical insights for both researchers and practitioners.

The HRC session did not affect MVIC force at any time point (i.e., post-exercise, or at 24 or 48 h), despite including a specific knee extension exercise, which contrasts with previous research that: (1) detected reductions in knee extensor MVIC force immediately after a bout of HRC [23] and (2) reported significant declines in maximal voluntary torque of the same muscle group following a resistance training session, an SIT protocol, and a concurrent exercise session (SIT followed by resistance exercises) [31]. However, in a study by Márquez et al. [23], the HRC protocol was specifically designed to induce fatigue, as it consisted of eight sets of each exercise, and in a study of Cross et al. [31] the resistance training session consisted of seven lower-body strength- and power-oriented exercises (e.g., back squats, lunges, deadlifts, squat jumps, etc.). In this regard, it is worth noting that our HRC session only contained one exercise that directly targeted the knee extensors (i.e., leg extension, performed in the first block), hence potentially allowing for a recovery of the neuromuscular function by the time MVIC force was assessed. This hypothesis is further supported by the fact that significant declines in MVIC force persisted at 24 h and 48 h only in the SIT conditions herein (characterized by greater lower-limb involvement due to the running-based activities). Indeed, concerning repeated-sprint efforts, researchers [24] have previously reported MVIC force declines exceeding 10% after a protocol comprising 12 maximal 30 m sprints interspersed with 30 s recovery periods. Furthermore, and consistently with the present data, a previous study demonstrated that the knee extensor MVIC force remained affected 48 h after a maximal running-based repeated-sprint protocol [32].

Notably, and contrary to our initial hypothesis, there was no impairment in force production post-exercise at 24 h or 48 h after the TCT session. Even though neuromuscular function has been shown to be perturbed following endurance exercise [33,34,35,36], MVIC force remained unchanged in the present research, despite TCT including 20 min of treadmill running after the resistance training portion of the workout. A possible reason for the phenomenon observed may be related to the fact that the duration of the running-based activity in TCT was considerably lower (i.e., 20 min) than in studies that found significant declines in MVIC force after prolonged aerobic exercise efforts of multiple hours (i.e., ranging from 5 h to ~22 h) [35,36]. From an applied perspective, given that fatigue is known to negatively affect performance and potentially increase injury risk [33,37,38,39], the present data suggest that careful planning of the training activities to be performed on the days following a SIT session is important, as neuromuscular function of the knee extensors may be expected to be impaired for up to 48 h.

With respect to the prevalence of peripheral fatigue, we observed significant decreases in tetanic force post-exercise after SIT (−24.6%), but not after HRC or TCT. These results are consistent with other studies that reported reductions in tetanic force after multiple sprint efforts [24,25,26,40], thereby confirming the occurrence of peripheral fatigue [24,25,26,31,41,42]. In their study, Goodall et al. [24] proposed that the contractile failure experienced following SIT may be related to disturbances in intramuscular metabolism that affect excitation–contraction coupling, to a reduced free calcium concentration or to an increased accumulation of inorganic phosphateresulting from the high blood lactate concentrations. Of note, no differences in tetanic force were found at 24 h or 48 h following SIT with respect to pre-exercise, despite the fact that MVIC force remained below pre-exercise values at those time points. It can be hypothesized that this decline in voluntary force production was due to the prevalence of low-frequency fatigue not noticeable with high-frequency stimulation [43], like the one used here (i.e., 100 Hz). Low-frequency fatigue is a long-lasting form of fatigue suggested to be caused by, among other factors, muscle fiber damage that may take several hours or even days to recover from and that is observed in response to low-frequency electrical stimulation (i.e., 10–40 Hz) [43]. Interestingly, a study by Venckunas et al. [44] demonstrated the prevalence of low-frequency fatigue (assessed as the ratio of stimulation torques at 20–100 Hz) 5 min, 1 h, and 24 h after different SIT formats (i.e., 6 × 5 s, 3 × 30 s and 3 × 60 s) in untrained young and older men and in endurance-trained cyclists. According to the authors, low-frequency fatigue was more pronounced in the untrained young cohort, possibly due to metabolic disturbances (e.g., release of calcium from the sarcoplasmic reticulum) caused by the SIT protocols [44]. Future studies should include both high- and low-frequency stimulation to better understand the origin and mechanisms of residual fatigue following HRC, SIT, and TCT.

According to previous research [34,38], peripheral fatigue is believed to play an important role in MVIC force reductions after high-intensity, short-duration exercise, whereas central fatigue may be more prominent after longer-duration efforts at lower intensities (e.g., low-force contractions or endurance exercise), as in TCT. Nevertheless, as discussed above, MVIC force did not significantly decrease post-exercise in this protocol, with no declines observed in tetanic force either. This occurrence seems to indicate that peripheral fatigue had no predominant role after TCT or HRC (as opposed to SIT). It is important to consider, however, that central fatigue mechanisms such as decreased neural drive [26,45] or cortical voluntary activation [46] have also been found following high-intensity exercise [26,42]. Hence, with the methodological approach used herein, it is not possible to exclude central fatigue as a potential origin of the neuromuscular fatigue observed following SIT. In fact, a previous investigation [47] reported that decreases in voluntary activation may occur after high-intensity efforts, even in the absence of important peripheral perturbations; hence, further research is warranted to better elucidate this issue.

Several limitations of the present study must be acknowledged. First, the HRC, SIT, and TCT sessions were dissimilar in design and thus imposed different neuromuscular and metabolic demands. Whilst this was a deliberate methodological choice in order to use sessions that might be likely to be used in practice, conclusions from the data must be made in this context. Nevertheless, the number of sets and repetitions were matched between protocols, ensuring parity in some aspects of session design. Second, the techniques used herein did not allow a clear determination of the contribution of central mechanisms [38]. In spite of the fact that the sole assessment of peripheral muscle function is not uncommon when examining fatigue in applied sport contexts [48,49], including after repeated-sprint exercise [50], the absence of techniques specifically designed to determine central fatigue responses in the present study (e.g., voluntary activation or central activation ratio measurements) warrants caution when interpreting these preliminary findings, as a concomitant central contribution to any MVIC force decline cannot be ruled out. Thirdly, rate of force development (RFD) was not assessed in the current study, despite being an important factor influencing physical function. Buckthorpe et al. [51] found that fatigue effects were more rapid in onset and marked for RFD than MVIC force, particularly during the initial 50 ms of the contraction, after a bout of repeated, high-force, explosive contractions. However, RFD assessment is methodologically complex and requires extensive participant familiarization to ensure valid and reliable data [52]. Moreover, according to Maffiuletti et al. [52], RFD measurements are highly sensitive to the type and characteristics of the equipment used, and commercially available isokinetic dynamometers (such as the one used here) are often too compliant for accurate assessment of RFD. Nevertheless, it must be acknowledged that RFD measurements would have provided additional, complementary insight into the presence of neuromuscular fatigue following the exercise protocols used in the current research.

A notable limitation is that the study might have been underpowered to detect small-to-moderate effects, as indicated by the sensitivity and power analyses. Additionally, the present data were obtained in trained, athletic males, so the results may not be transferrable to females, athletes of greater training age or performance caliber, or aged or clinical populations and must be interpreted with caution. Although strict criteria in terms of training experience were established in the current investigation to reduce heterogeneity in participants’ background (e.g., resistance vs. endurance training), which could potentially affect the results, it should be acknowledged that some degree of interindividual variability likely remained and may have influenced the observed responses. Thus, further research is required to better understand the acute impact of these training sessions with greater sample sizes and across different population cohorts. Finally, it would be interesting to study the short- and long-term adaptations to HRC, SIT, and TCT to determine whether they are suitable options to improve neuromuscular and cardiovascular parameters and body composition in different types of population. Practitioners might then use information relating to the fatigue induced by single sessions as well as the adaptations evoked by longer-term training to make informed choices as to exercise program designs.

## 5. Conclusions

The present results indicate that a bout of HRC and TCT induced little or no fatigue of the knee extensors, contrary to a single session of SIT, in which impairments in neuromuscular function were identified up to 48 h. Peripheral fatigue mechanisms seemed to prevail following the repeated-sprint exercise protocol in comparison with the HRC and TCT protocols, most probably due to the considerably higher exercise intensity (i.e., all-out effort). From an applied perspective, practitioners should consider the demands and time course of fatigue/recovery after different modalities of concurrent training, as these may have implications in terms of acute performance and injury risk. HRC or TCT should better be utilized when faster recoveries are important (e.g., in-season or preseason in team sports). However, the former protocol is more suitable when limited time is available. In contrast, SIT may be a good option if the objective is to perform running-based concurrent training in contexts in which recovery is not a priority (e.g., off-season).

## Figures and Tables

**Table 1:** The descriptive data for the different variables at each time-point following the HRC, SIT and TCT protocols.

		HRC	SIT	TCT
MVIC force (N)	Pre-exercise	640 ± 95	656 ± 93	654 ± 97
	Post-exercise	610 ± 123	559 ± 78	582 ± 95
	24 h	633 ± 116	555 ± 75	614 ± 96
	48 h	641 ± 118	543 ± 64	620 ± 93
Tetanic force (N)	Pre-exercise	284 ± 84	280 ± 49	289 ± 45
	Post-exercise	297 ± 62	211 ± 58	297 ± 66
	24 h	312 ± 64	263 ± 97	277 ±87
	48 h	268 ± 55	289 ± 100	321 ± 82

Mean values ± standard deviation. HRC = high-resistance circuit training; SIT = sprint interval training; TCT = traditional concurrent training; MVIC = maximum voluntary isometric contraction.

## Data Availability

The data that support the findings of this study are available from the corresponding author upon reasonable request.

## References

[1] Karcher C., Buchheit M. (2014). On-court demands of elite handball, with special reference to playing positions. Sports Med..

[2] Bloomfield J., Polman R., O’Donoghue P. (2007). Physical demands of different positions in FA Premier League soccer. J. Sports Sci. Med..

[3] Fyfe J.J., Bishop D.J., Stepto N.K. (2014). Interference between concurrent resistance and endurance exercise: Molecular bases and the role of individual training variables. Sports Med..

[4] Fyfe J.J., Loenneke J.P. (2018). Interpreting adaptation to concurrent compared with single-mode exercise training: Some methodological considerations. Sports Med..

[5] Deschenes M.R., Kraemer W.J. (2002). Performance and physiologic adaptations to resistance training. Am. J. Phys. Med. Rehabil..

[6] Suchomel T.J., Nimphius S., Stone M.H. (2016). The importance of muscular strength in athletic performance. Sports Med..

[7] Coffey V.G., Hawley J.A. (2017). Concurrent exercise training: Do opposites distract?. J. Physiol..

[8] Guo Z., Li M., Cai J., Gong W., Liu Y., Liu Z. (2023). Effect of High-Intensity Interval Training vs. Moderate-Intensity Continuous Training on Fat Loss and Cardiorespiratory Fitness in the Young and Middle-Aged a Systematic Review and Meta-Analysis. Int. J. Environ. Res. Public Health.

[9] Reeves N.D., Narici M.V., Maganaris C.N. (2004). Effect of resistance training on skeletal muscle-specific force in elderly humans. J. Appl. Physiol..

[10] Kelley D.E., He J., Menshikova E.V., Ritov V.B. (2002). Dysfunction of mitochondria in human skeletal muscle in type 2 diabetes. Diabetes.

[11] Helgerud J., Rodas G., Kemi O., Hoff J. (2011). Strength and endurance in elite football players. Int. J. Sports Med..

[12] Jones T.W., Smith A., Macnaughton L.S., French D.N. (2016). Strength and conditioning and concurrent training practices in elite rugby union. J. Strength Cond. Res..

[13] Panissa V.L., Fukuda D.H., Parmezzani S., Campos E., Rossi F., Franchini E., Lira F. (2018). Maximum Strength Development and Volume-Load during Concurrent High Intensity Intermittent Training Plus Strength or Strength-Only Training. J. Sports Sci. Med..

[14] Häkkinen K., Alen M., Kraemer W., Gorostiaga E., Izquierdo M., Rusko H., Mikkola J., Häkkinen A., Valkeinen H., Kaarakainen E. (2003). Neuromuscular adaptations during concurrent strength and endurance training versus strength training. Eur. J. Appl. Physiol..

[15] MacInnis M.J., Gibala M.J. (2017). Physiological adaptations to interval training and the role of exercise intensity. J. Physiol..

[16] Burgomaster K.A., Howarth K.R., Phillips S.M., Rakobowchuk M., MacDonald M.J., McGee S.L., Gibala M.J. (2008). Similar metabolic adaptations during exercise after low volume sprint interval and traditional endurance training in humans. J. Physiol..

[17] Gibala M.J., Little J.P., Van Essen M., Wilkin G.P., Burgomaster K.A., Safdar A., Raha S., Tarnopolsky M.A. (2006). Short-term sprint interval versus traditional endurance training: Similar initial adaptations in human skeletal muscle and exercise performance. J. Physiol..

[18] Kinnunen J.-V., Piitulainen H., Piirainen J.M. (2019). Neuromuscular Adaptations to Short-Term High-Intensity Interval Training in Female Ice-Hockey Players. J. Strength Cond. Res..

[19] Muñoz-Martínez F.A., Rubio-Arias J.A., Ramos-Campo D.J., Alcaraz P.E. (2017). Effectiveness of resistance circuit-based training for maximum oxygen uptake and upper-body one-repetition maximum improvements: A systematic review and meta-analysis. Sports Med..

[20] Alcaraz P.E., Perez-Gomez J., Chavarrias M., Blazevich A.J. (2011). Similarity in adaptations to high-resistance circuit vs. traditional strength training in resistance-trained men. J. Strength Cond. Res..

[21] Romero-Arenas S., Blazevich A.J., Martínez-Pascual M., Pérez-Gómez J., Luque A.J., López-Román F.J., Alcaraz P.E. (2013). Effects of high-resistance circuit training in an elderly population. Exp. Gerontol..

[22] Martínez-Guardado I., Ramos-Campo D.J., Olcina G.J., Rubio-Arias J.A., Chung L.H., Marín-Cascales E., Alcaraz P.E., Timón R. (2019). Effects of high-intensity resistance circuit-based training in hypoxia on body composition and strength performance. Eur. J. Sport Sci..

[23] Márquez G., Romero-Arenas S., Marín-Pagán C., Vera-Ibañez A., FernáNdez Del Olmo M., Taube W. (2017). Peripheral and central fatigue after high intensity resistance circuit training. Muscle Nerve.

[24] Goodall S., Charlton K., Howatson G., Thomas K. (2015). Neuromuscular fatigability during repeated-sprint exercise in male athletes. Med. Sci. Sports Exerc..

[25] Perrey S., Racinais S., Saimouaa K., Girard O. (2010). Neural and muscular adjustments following repeated running sprints. Eur. J. Appl. Physiol..

[26] Pearcey G.E., Murphy J.R., Behm D.G., Hay D.C., Power K.E., Button D.C. (2015). Neuromuscular fatigue of the knee extensors during repeated maximal intensity intermittent-sprints on a cycle ergometer. Muscle Nerve.

[27] Wasserman K., Beaver W.L., Whipp B.J. (1990). Gas exchange theory and the lactic acidosis (anaerobic) threshold. Circulation.

[28] Freitas T.T., Calleja-González J., Alarcón F., Alcaraz P.E. (2016). Acute effects of two different resistance circuit training protocols on performance and perceived exertion in semiprofessional basketball players. J. Strength Cond. Res..

[29] Alcaraz P.E., Sanchez-Lorente J., Blazevich A.J. (2008). Physical performance and cardiovascular responses to an acute bout of heavy resistance circuit training versus traditional strength training. J. Strength Cond. Res..

[30] Hopkins W.G., Marshall S.W., Batterham A.M., Hanin J. (2009). Progressive statistics for studies in sports medicine and exercise science. Med. Sci. Sports Exerc..

[31] Cross R., Lovell R., Marshall P.W., Siegler J. (2021). Acute Neuromuscular Response to Team Sports–specific Running, Resistance, and Concurrent Training: A Cross-over Study. Med. Sci. Sports Exerc..

[32] Baumert P., Temple S., Stanley J., Cocks M., Strauss J.A., Shepherd S.O., Drust B., Lake M.J., Stewart C.E., Erskine R.M. (2021). Neuromuscular fatigue and recovery after strenuous exercise depends on skeletal muscle size and stem cell characteristics. Sci. Rep..

[33] Ribeiro N., Ugrinowitsch C., Panissa V.L.G., Tricoli V. (2019). Acute effects of aerobic exercise performed with different volumes on strength performance and neuromuscular parameters. Eur. J. Sport Sci..

[34] Twomey R., Aboodarda S.J., Kruger R., Culos-Reed S.N., Temesi J., Millet G.Y. (2017). Neuromuscular fatigue during exercise: Methodological considerations, etiology and potential role in chronic fatigue. Clin. Neurophysiol..

[35] Place N., Lepers R., Deley G., Millet G.Y. (2004). Time course of neuromuscular alterations during a prolonged running exercise. Med. Sci. Sports Exerc..

[36] Temesi J., Arnal P.J., Rupp T., Féasson L., Cartier R., Gergelé L., Verges S., Martin V., Millet G.Y. (2015). Are females more resistant to extreme neuromuscular fatigue?. Med. Sci. Sports Exerc..

[37] Lewis M. (2018). It’s a Hard-Knock Life: Game Load, Fatigue, and Injury Risk in the National Basketball Association. J. Athl. Train..

[38] Carroll T.J., Taylor J.L., Gandevia S.C. (2016). Recovery of central and peripheral neuromuscular fatigue after exercise. J. Appl. Physiol..

[39] Knicker A.J., Renshaw I., Oldham A.R., Cairns S.P. (2011). Interactive processes link the multiple symptoms of fatigue in sport competition. Sports Med..

[40] Lloria-Varella J., Koral J., Ravel A., Féasson L., Murias J.M., Busso T. (2023). Neuromuscular and autonomic function is fully recovered within 24 h following a sprint interval training session. Eur. J. Appl. Physiol..

[41] Glaister M. (2005). Multiple sprint work. Sports Med..

[42] Collins B.W., Pearcey G.E., Buckle N.C., Power K.E., Button D.C. (2018). Neuromuscular fatigue during repeated sprint exercise: Underlying physiology and methodological considerations. Appl. Physiol. Nutr. Metab..

[43] Keeton R.B., Binder-Macleod S.A. (2006). Low-frequency fatigue. Phys. Ther..

[44] Venckunas T., Krusnauskas R., Snieckus A., Eimantas N., Baranauskiene N., Skurvydas A., Brazaitis M., Kamandulis S. (2019). Acute effects of very low-volume high-intensity interval training on muscular fatigue and serum testosterone level vary according to age and training status. Eur. J. Appl. Physiol..

[45] Girard O., Mendez-Villanueva A., Bishop D. (2011). Repeated-sprint ability—Part I. Sports Med..

[46] Fernandez-del-Olmo M., Rodriguez F., Marquez G., Iglesias X., Marina M., Benitez A., Vallejo L., Acero R. (2013). Isometric knee extensor fatigue following a Wingate test: Peripheral and central mechanisms. Scand. J. Med. Sci. Sports.

[47] Morel B., Lapole T., Liotard C., Hautier C. (2019). Critical peripheral fatigue thresholds among different force-velocity conditions: An individual-based model approach. Front. Physiol..

[48] Rampinini E., Martin M., Davide F., Bosio A., Azzolini M., Riggio M., Maffiuletti N.A. (2022). Peripheral muscle function during repeated changes of direction in professional soccer players. Eur. J. Appl. Physiol..

[49] Ferioli D., Rampinini E., Bosio A., La Torre A., Maffiuletti N.A. (2019). Peripheral muscle function during repeated changes of direction in basketball. Int. J. Sports Physiol. Perform..

[50] Rampinini E., Connolly D.R., Ferioli D., La Torre A., Alberti G., Bosio A. (2016). Peripheral neuromuscular fatigue induced by repeated-sprint exercise: Cycling vs. running. J. Sports Med. Phys. Fit..

[51] Buckthorpe M., Pain M.T., Folland J.P. (2014). Central fatigue contributes to the greater reductions in explosive than maximal strength with high-intensity fatigue. Exp. Physiol..

[52] Maffiuletti N.A., Aagaard P., Blazevich A.J., Folland J., Tillin N., Duchateau J. (2016). Rate of force development: Physiological and methodological considerations. Eur. J. Appl. Physiol..

